# Verfügbarkeit von Patienteninformationen in der Notaufnahme: Wunsch und Wirklichkeit

**DOI:** 10.1007/s00063-021-00881-6

**Published:** 2021-10-28

**Authors:** J. Born, A. Bohn, P. Kümpers, C. Juhra

**Affiliations:** 1 Stabsstelle Telemedizin, Universitätsklinikum Münster, Hüfferstraße 73–79, 48149 Münster, Deutschland; 2 Ärztliche Leitung Rettungsdienst, Stadt Münster, Feuerwehr, Münster, Deutschland; 3 Klinik für Anästhesiologie, operative Intensivmedizin und Schmerztherapie, Universitätsklinikum Münster, Münster, Deutschland; 4 Medizinische Klinik D für Allg. Innere Medizin und Notaufnahme sowie Nieren- und Hochdruckkrankheiten und Rheumatologie, Universitätsklinikum Münster, Münster, Deutschland

**Keywords:** Gesundheitsinformationsaustausch, Telematik, E‑Health, Notfalldatensatz, Notfallmedizin, Health information exchange, Telemedicine, eHealth, Medical emergency dataset, Emergency medicine

## Abstract

**Hintergrund:**

Gerade in der Notfallmedizin ist der schnelle Zugriff auf Informationen anderer Leistungserbringer von besonderer Bedeutung, da die Patienten oftmals unbekannt sind und Behandlungsentscheidungen zeitnah getroffen werden müssen.

**Ziel der Arbeit:**

Die Studie zielt darauf ab, herauszufinden, mit welchen Herausforderungen die Notaufnahmen bei der Informationsbeschaffung konfrontiert sind, wie hoch der erwartete Nutzen eines einfacheren Informationszugangs ist und welche Informationen zur Patientenvorgeschichte dort am dringendsten benötigt werden.

**Material und Methoden:**

Durchgeführt wurde eine deutschlandweite Online-Befragung unter in Notaufnahmen tätigem medizinischem Personal. 181 Fragebögen wurden vollständig ausgefüllt und konnten in die Datenanalyse einbezogen werden.

**Ergebnisse:**

Insgesamt 77,9 % des befragten Notaufnahmepersonals bewertete es als schwierig oder sehr schwierig, im Rahmen der Patientenversorgung an klinische Informationen von außen zu gelangen. Im Durchschnitt benötigen die Befragungsteilnehmer ihren Schätzungen zufolge rund 47 min, um an Informationen zu einem Patienten zu gelangen. 99,4 % gehen davon aus, dass die Patientenversorgung von einem einfacheren und schnelleren Informationsaustausch profitieren würde. Als wichtigste Datenelemente wurden Medikationslisten, Entlassungsbriefe und Informationen zu Vorerkrankungen sowie Allergien eingestuft.

**Diskussion:**

In Anbetracht des erheblichen Aufwandes für die Informationsbeschaffung bei Notfallpatienten besteht ein dringlicher Handlungsbedarf. Digitale Lösungen wie der gerade eingeführte Notfalldatensatz können bei flächendeckender Verbreitung hier einen Mehrwert für die klinische Notfallversorgung bieten.

## Abstract (en)

**Background:**

Fast access to information from other healthcare service providers is particularly important in emergency medicine, as the patients are often unknown and treatment decisions have to be made promptly.

**Objectives:**

The study aims to identify the challenges that emergency departments face in obtaining information on patient history, the expected benefits of easier access to information and which information is most urgently needed.

**Materials and methods:**

An online survey throughout Germany was carried out among medical staff working in emergency departments. In all, 181 questionnaires were fully completed and could be included in the data analysis.

**Results:**

Of the respondents, 77.9% said it was difficult or very difficult to receive external data at the point of patient care. The survey participants estimate that they need an average of around 47 min to obtain information about one patient. 99.4% believe that patient care would benefit from an easier and faster information exchange. Medication lists, discharge letters, information on previous illnesses and allergies were classified as the most important data elements.

**Conclusions:**

There is an urgent need for action with regard to the considerable effort involved in obtaining information on emergency patients. Digital solutions such as the recently introduced emergency data set can offer additional value for clinical emergency care if they are widely used.

In der Notfallversorgung müssen komplexe Behandlungsentscheidungen oftmals unter hohem Zeitdruck getroffen werden [1]. Erschwerend hinzu kommt, dass der Zustand von Notfallpatienten nicht immer eine anamnestische Befragung erlaubt. Der schnelle Zugriff auf Informationen anderer Leistungserbringer zur Patientenvorgeschichte wie Vorerkrankungen, Medikamenteneinnahmen oder Allergien ist daher in der Notfallmedizin von besonderer Relevanz [2].

Mit der zunehmenden Arbeitsbelastung in deutschen Notaufnahmen [3] gewinnt die rasche und unkomplizierte Verfügbarkeit von notfallrelevanten Patienteninformationen in diesem Bereich noch zusätzlich an Bedeutung. Lokale Untersuchungen in Münster und New York weisen darauf hin, dass sich beim Fehlen von Vorbefunden die Anamnesedauer in Notaufnahmen deutlich verlängert [4] und Versuche, Informationen zur Patientenvorgeschichte von externen Leistungserbringern zu beziehen, meist sehr zeitintensiv und schwierig sind [5]. So bleiben die Bemühungen um externe Informationen laut zwei Drittel der New Yorker Befragten in mehr als der Hälfte der Fälle erfolglos [5]. Gründe für die oftmals schwierige Informationsbeschaffung sind unter anderem ein fragmentiertes Gesundheitssystem, papierbasierte Prozesse sowie eine unzureichende Nutzung von Datenstandards und einheitlichen Terminologien [6].

Stiell et al. [7] konnten in einer Studie mit 1002 Patientenkontakten in einer kanadischen Notaufnahme zeigen, dass bei rund 32 % der Patientenkontakte die Informationen zur medizinischen Vorgeschichte unvollständig waren. Bei älteren Patienten und bei Patienten mit ernsthaften chronischen Vorerkrankungen traten entsprechende Informationslücken zudem signifikant häufiger auf. Es kann somit postuliert werden, dass mit dem starken Anstieg von älteren Patienten in deutschen Notaufnahmen [8] die Dringlichkeit eines funktionierenden digitalen Austausches von Gesundheitsinformationen noch weiter zunimmt.

Ein erster Schritt wurde 2020 in Deutschland mit dem Beginn der Etablierung des Notfalldatensatzes (NFD) und des Datensatzes persönliche Erklärungen (DPE) gemacht, welche zu den prioritären medizinischen Anwendungen der elektronischen Gesundheitskarte (eGK) zählen [9]. Künftig sollen bundesweit auf Wunsch der Versicherten notfallrelevante Informationen wie chronische Erkrankungen und Allergien durch autorisierte Akteure des Gesundheitswesens (u. a. Hausärzte) auf der eGK gespeichert und im Notfall von anderen Ärzten oder deren berufsmäßigen Gehilfen mittels eines elektronischen Ausweises abgerufen werden können [2, 10]. Aufgrund des limitierten Speicherplatzes der eGK ist der Inhalt des Notfalldatensatzes bisher auf Textinformationen begrenzt, elektronische Dokumente wie EKG-Befunde, Röntgen- oder CT-Bilder können nicht hinterlegt werden [11].

In den Jahren 2021 und 2022 soll die Digitalisierung des Informationsaustausches im Gesundheitswesen unter anderem mit der Einführung der elektronischen Patientenakte (ePA) sowie des elektronischen Rezeptes noch weiter vorangetrieben werden [9]. Inmitten der vielfältigen digitalen Anwendungen, die derzeit mit dem Ausbau der Telematikinfrastuktur (TI) auf den Weg gebracht werden, sollten die konkreten Informationsbedürfnisse der späteren Anwender sowie der praktische Nutzen stets im Blick behalten werden.

Ein zentrales Ziel dieser Studie war es daher, die Frage zu beantworten, welche Informationen zur Patientenvorgeschichte aus Sicht des in klinischen Notaufnahmen tätigen medizinischen Personals am dringendsten benötigt werden und in welcher Form diese zur Verfügung gestellt werden sollten. Außerdem wurde nach Kenntnis der Autoren mit der vorliegenden Befragung erstmals deutschlandweit erhoben, mit welchen Herausforderungen die Notfallambulanzen gegenwärtig bei der Informationsbeschaffung konfrontiert sind, mit welchem Aufwand dies verbunden ist und wie hoch der erwartete Nutzen eines verbesserten Zugriffs auf Informationen zur medizinischen Vorgeschichte ist.

## Methodik

Zur Beantwortung der Fragestellungen wurde eine deutschlandweite Online-Befragung unter in der klinischen Notfallversorgung tätigem medizinischem Personal durchgeführt. Die Befragungsteilnehmer wurden über den E‑Mail-Verteiler der Deutschen Gesellschaft für interdisziplinäre Notfall- und Akutmedizin (DGINA) rekrutiert, welcher am Versanddatum 1530 Personen umfasste, von denen nach Auskunft der DGINA etwa die Hälfte in der klinischen Notfallversorgung tätig war und damit der Zielgruppe der Befragung angehörte. Der erste Aufruf zur Befragungsteilnahme wurde am 18.07.2019 mit dem monatlichen Newsletter der DGINA versendet und umfasste kurze Informationen zu Zielen, Zielgruppe und Umfang der Befragung sowie einen Hyperlink, der direkt zu dem mit der Online-Umfrage-Applikation LimeSurvey (LimeSurvey GmbH, Hamburg, https://www.limesurvey.org/de/) umgesetzten Online-Fragebogen führte. Für Personen, die den Link zu Befragung erhalten hatten, bestand die Möglichkeit, diesen an weitere Kollegen in der klinischen Notfallversorgung weiterzuleiten. Nach drei Wochen wurde in dem nächsten Newsletter der DGINA noch einmal an die Befragung erinnert. Außerdem wurde ergänzend auch über die Facebookgruppe der Young DGINA, eine notfallmedizinisch interessierte Gruppe aus Assistenzärzten, Pflege- und Rettungsdienst in Ausbildung sowie Medizinstudierenden, auf die Befragung aufmerksam gemacht. Die Möglichkeit zur Befragungsteilnahme endete am 30.09.2019.

Die Teilnahme an der Befragung erfolgte freiwillig und erforderte keinen Einbezug von Patienten. Da außerdem keine Daten, die Rückschlüsse auf die Identität der jeweiligen Befragungsperson zulassen oder anderweitig sensible Informationen erhoben wurden, konnte auf ein Ethikvotum verzichtet werden.

### Befragungsinstrument

Zur Beantwortung der Fragen wurde ein Fragebogen selbst entwickelt, welcher die Themenbereiche klinischer Tätigkeitsbereich und Versorgungsprozess, Wahrnehmung der Telematikinfrastruktur in Deutschland, Verfügbarkeit und wahrgenommener Nutzen von Informationen zur Patientenvorgeschichte sowie Art der gewünschten klinischen Informationen abdeckt. Um eine internationale Vergleichbarkeit der Ergebnisse zu ermöglichen, orientieren sich die Fragen der letztgenannten Themenbereiche an einem bereits erprobten Befragungsinstrument, welches von Shapiro et al. [5] für eine Studie zum Gesundheitsinformationsaustausch in New Yorker Notaufnahmen entwickelt und den Autoren dieser Studie zur Verfügung gestellt wurde. Die ausgewählten Fragen wurden ins Deutsche übertragen und modifiziert, um sie an die spezifischen Gegebenheiten des deutschen Gesundheitssystems anzupassen.

Zur Überprüfung der Funktionsfähigkeit und Verständlichkeit der Frageitems, wurde ein Prätest mit zwölf Personen aus unterschiedlichen Fachrichtungen und Funktionsbereichen innerhalb der klinischen Notfallversorgung durchgeführt. Außerdem wurde der Fragebogen der damaligen AG Wissenschaft der DGINA zur Begutachtung vorgelegt. Auf Grundlage der erhaltenen Rückmeldungen wurde der Fragebogen anschließend modifiziert.

Die finale Version des Fragebogens beinhaltet insgesamt 21 Items, davon 15 geschlossene, zwei halboffene und vier offene Fragen. Der Fragebogen ist bei der Erstautorin auf Anfrage erhältlich.

### Datenaufbereitung und Auswertung

Eine erste statistische Auswertung der Befragungsdaten erfolgte mit Hilfe der in LimeSurvey integrierten Statistikfunktion. Anschließend wurden die Befragungsdaten für weitergehende Analysen in Microsoft Excel exportiert. Zur Auswertung der offenen oder halboffenen Fragen wurden die dort generierten Freitextangaben zunächst aufgelistet und anschließend in Antwortkategorien zusammengefasst. In die Auswertung eingeschlossen wurden nur Fragebögen, bei denen die Pflichtfragen vollständig beantwortet wurden.

## Ergebnisse

Die Online-Umfrage wurde im Befragungszeitraum insgesamt 372-mal aufgerufen. 181 Fragebögen wurden vollständig ausgefüllt und konnten in die statistische Datenanalyse einbezogen werden.

Von den 181 Befragungsteilnehmern waren zum Zeitpunkt der Befragung rund 80 % in einer interdisziplinären Notaufnahme tätig. Die übrigen Befragten nannten als überwiegenden Tätigkeitsbereich die chirurgische Notaufnahme, die internistische Notaufnahme, die Stroke Unit oder sonstige Bereiche (Tab. 1), darunter mit zwei Nennungen die pädiatrische Notaufnahme. Im Hinblick auf die berufliche Funktion gehörte mit über 70 % die Mehrheit der Befragungsteilnehmer dem ärztlichen Dienst an, weitere Teilnehmer waren als Pflegefachkraft (14 Personen) bzw. Pflegefachkraft in leitender Position (27 Personen) oder als medizinische Fachangestellte (2 Personen) tätig (Tab. 1). Unter Sonstiges wurden als weitere berufliche Funktionen u. a. Notfallpflegefachkraft, Notfallsanitäter und Physician Assistant angegeben.

**Table 1:** 

	Fallzahl (*n*)	Relative Häufigkeit (%)
*Überwiegender klinischer Tätigkeitsbereich*		
Interdisziplinäre Notaufnahme	145	80,1
Internistische Notaufnahme	11	6,1
Chirurgische Notaufnahme	13	7,2
Stroke Unit	1	0,6
Chest Pain Unit	0	0,0
Sonstiges	11	6,1
*Berufliche Funktion*		
Pflegefachkraft	14	7,7
Pflegefachkraft in leitender Position	27	14,9
Medizinischer Fachangestellter	2	1,1
Assistenzarzt	31	17,1
Facharzt	6	3,3
Oberarzt	42	23,2
Chefarzt	48	26,5
Sonstiges	14	6,1

### Aktuelle Herausforderungen und Aufwand

**Figure 1:**
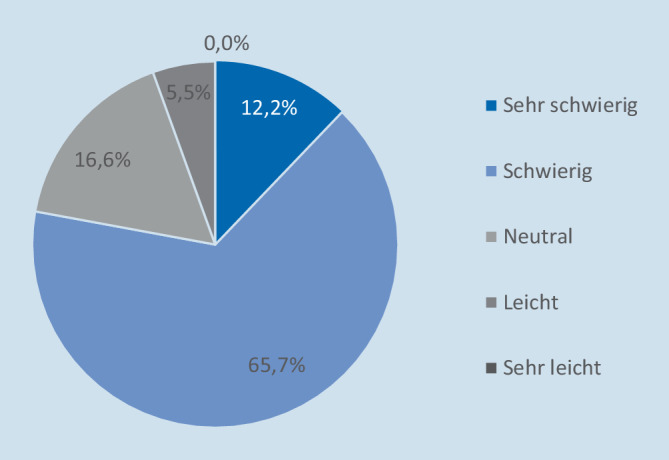

Auf die Frage, bei wie vielen Patienten versucht wird, an klinische Informationen von externen Leistungserbringern zu gelangen, antworteten 51,9 % des befragten Notaufnahmepersonals, dass dies bei mehr als einem Viertel der Patienten der Fall sei, darunter 18,8 %, bei denen es sogar mehr als die Hälfte der Patienten betraf. Wenn externe Informationen zur Vervollständigung der Anamnese bei Notfallpatienten benötigt werden, werden diese in der Regel noch während der Versorgung in der Notaufnahme angefragt, so die mehrheitliche Äußerung der Befragungsteilnehmer (83,4 %). Jedoch gaben rund 52 % der Befragten an, dass nicht mehr als die Hälfte ihrer Versuche, klinische Informationen von externen Leistungserbringern zu erhalten, erfolgreich sind. Gleichzeitig bewerteten 77,9 % es als schwierig oder sehr schwierig, im Rahmen der Patientenversorgung an relevante klinische Informationen von außen zu gelangen (Abb. 1). Wie aus den ergänzenden Freitextanmerkungen hervorgeht, stellen insbesondere hohe Datenschutzanforderungen sowie die begrenzten Öffnungszeiten von Arztpraxen Herausforderungen für das Notaufnahmepersonal bei der Informationsbeschaffung dar. Im Durchschnitt benötigen die Befragungsteilnehmer ihren Schätzungen zufolge rund 47 min (SD: ± 40,5 min), um von externen Leistungserbringern Informationen zu einem Patienten einzuholen.

### Erwarteter Nutzen

**Figure 2:**
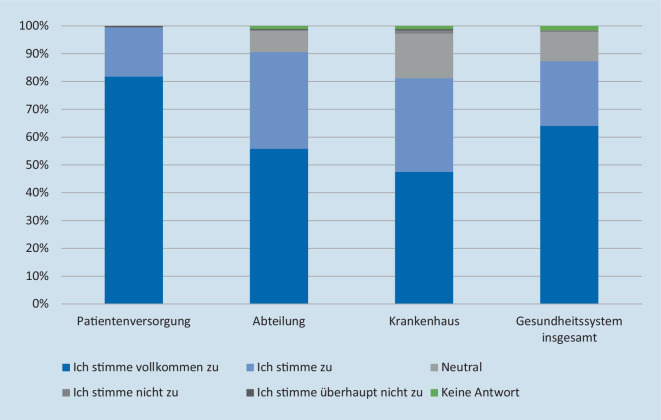

Mit jeweils über 80 % Zustimmung geht die Mehrheit der Befragten davon aus, dass sowohl die Patientenversorgung als auch ihre jeweilige Abteilung, ihr Krankenhaus sowie das Gesundheitssystem insgesamt davon profitieren würden, wenn klinische Informationen aus anderen Krankenhäusern zum Zeitpunkt der Patientenversorgung schnell und einfach zur Verfügung stünden (Abb. 2). Auf die Frage, wie viele ihrer Patienten konkret von einem schnellen Informationszugang profitieren würden, antworteten 65,7 %, dass dies bei mehr als der Hälfte ihrer Patienten der Fall sei. Damit einhergehend denken rund 96 %, dass ein verbesserter Zugriff auf Patientenvorinformationen die Effizienz der klinischen Versorgung vergrößern oder stark vergrößern würde. Von den Befragten erwarten 84,5 % als weiteren Effekt eine Verringerung der Anzahl der durchzuführenden Untersuchungen und 80,7 % die Reduzierung medizinischer Fehler. Außerdem rechnen rund 59 % damit, dass die Zeit bis zur Entscheidung zur Weiterbehandlung bzw. Verlegung einzelner Patienten verkürzt würde.

Der Großteil des befragten Notaufnahmepersonals ist der Meinung, dass die erwarteten Vorteile die möglichen Risiken eines einfacheren Zugangs zu klinischen Informationen von externen Leistungserbringern deutlich überwiegen (68,5 %) bzw. überwiegen (23,8 %). Wenn verfügbar, würden 85,6 % gerne eine kurze Zusammenfassung der Patientenvorgeschichte lesen, bevor sie ihre Patienten sehen.

### Art der gewünschten klinischen Informationen

**Figure 3:**
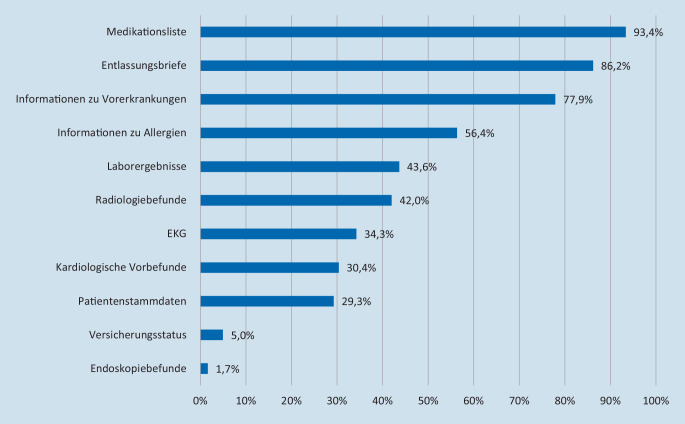

Wie Abb. 3 zeigt, wurden die Befragungsteilnehmer zur Erfassung ihrer Informationsprioritäten gebeten, aus einer Liste von elf Datenelementen die aus ihrer Sicht wichtigsten fünf auszuwählen und entsprechend der beigemessenen Relevanz zu sortieren. Mit Abstand am häufigsten unter die wichtigsten fünf Datenelemente gewählt wurden die Medikationsliste (93,4 %), die Entlassungsbriefe (86,2 %) und die Informationen zu Vorerkrankungen (77,9 %). Dabei wurden die Entlassungsbriefe mit rund 35 % am häufigsten auf den ersten Rang gesetzt, gefolgt von der Medikationsliste mit knapp 26 %. Über die elf abgefragten Datenelemente hinaus (Abb. 3) wurden innerhalb von Freitextangaben außerdem Patientenverfügung bzw. Vorsorgevollmacht (57 Nennungen), Kontaktdaten von Angehörigen oder Betreuern (40 Nennungen) sowie Versorgungssituation bzw. Pflegestatus (25 Nennungen) vielfach als nützliche Informationen bei der Patientenversorgung benannt.

**Figure 4:**
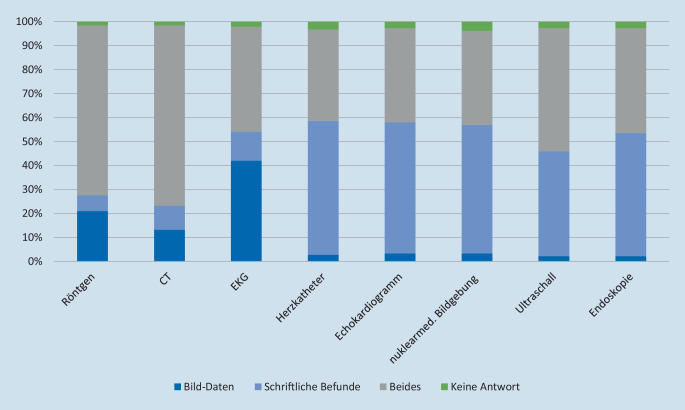

Auf die Frage, ob sie lieber schriftliche Befunde oder Bilddaten oder beides zusammen erhalten würden, gaben jeweils knapp über 50 % der Befragten an, dass sie im Falle von Herzkatheteruntersuchungen, Echokardiographie, nuklearmedizinsicher Bildgebung und Endoskopie schriftliche Befunde bevorzugen würden (Abb. 4). Eine Kombination aus schriftlichen Befunden und Bilddaten wurde für Röntgenbefunde von 70,7 % der Befragungsteilnehmer bevorzugt, für Computertomographie (CT) von 75,1 % und für Ultraschall von 51,4 %. 43,6 % würden auch zu einer Elektrokardiographie (EKG) gerne Bilddaten in Kombination mit einem schriftlichen Befund übermittelt bekommen, während für 42 % die Übermittlung nur von Bilddaten ausreichend wäre.

### Wahrnehmung der Telematikinfrastruktur

Zu der in Deutschland derzeit im Aufbau befindlichen Telematikinfrastruktur befragt, gaben rund 78 % des befragten Notaufnahmepersonals an, von dieser bereits gehört zu haben. Rund einem Fünftel war die TI zuvor jedoch noch kein Begriff. Von den medizinischen Fachanwendungen der TI war die ePA unter den Befragungsteilnehmern mit 84 % die bekannteste. An zweiter Stelle folgte mit einem Bekanntheitsgrad von 63 % der elektronische Medikationsplan und auf Rang drei das Versichertenstammdatenmanagement mit 53 %. Mit dem Begriff Notfalldaten-Management konnten 46,4 % der Befragten etwas anfangen, bei der elektronischen Verordnung waren es 44,8 %. Insgesamt 7,2 % der befragten Personen gaben an, dass ihnen keine der fünf genannten medizinischen Fachanwendungen ein Begriff sei.

Im Hinblick auf die mutmaßlichen ökonomischen Auswirkungen der TI schätzen knapp 52 % der Befragten, dass die Einsparungen die Kosten für den Aufbau übersteigen werden. Dass sich die Kosten für den Aufbau mit den Einsparungen die Waage halten werden, vermutet rund ein Viertel, während 14,4 % der Befragungsteilnehmer davon ausgehen, dass die Kosten für den Aufbau der TI die Einsparungen übersteigen werden.

## Diskussion

Die Befragungsergebnisse zeigen, dass in deutschen Notaufnahmen die Beschaffung von Informationen zur Patientenvorgeschichte von externen Leistungserbringern sehr oft schwierig und zeitaufwändig ist. Der durchschnittliche geschätzte Zeitaufwand fällt zwar etwas geringer aus als in der New Yorker Studie von 2007, wo dieser sogar auf 66 min beziffert wurde [5], doch bedeutet auch ein durchschnittlicher Aufwand von 47 min pro Patient eine deutliche Mehrbelastung des Notaufnahmepersonals. Wenn Versuche der Informationsgewinnung zu lange dauern oder zum Teil sogar ganz erfolglos bleiben und deshalb zeitkritische Behandlungsentscheidungen getroffen werden müssen, ohne dass notfallrelevante Patienteninformationen wie z. B. zu Medikamenteneinnahmen oder -allergien vorliegen, kann dies zudem ein ernstzunehmendes Sicherheitsrisiko in der Versorgung von Notfallpatienten darstellen. In einer Beobachtungsstudie von Lorsbach et al. [4] zeigte sich, dass bei acht von 94 Patienten die Therapie- und/oder Diagnostikentscheidungen anders ausgefallen wären, wenn alle Informationen zur Patientenvorgeschichte bereits zu Beginn der Behandlung in der Notaufnahme vorgelegen hätten. Auch andere Studienergebnisse mit größeren Fallzahlen weisen darauf hin, dass Informationen zur Patientenvorgeschichte in der Notfallversorgung eine hohe Relevanz für Diagnostik- und Therapieentscheidungen haben können [7].

Den in dieser Studie aufgezeigten Schwierigkeiten beim Informationsaustausch gegenüber steht ein hoher erwarteter Nutzen eines schnelleren Datentransfers, nicht nur für den einzelnen Patienten, sondern auch für das Gesundheitssystem insgesamt. Diese Einschätzung der Befragten deckt sich mit den Ergebnissen eines systematischen Reviews von Menachemi et al. [12], wonach ein digitaler Gesundheitsinformationsaustausch sich positiv auf die Patientensicherheit auswirkt und gleichzeitig die Zahl der Doppeluntersuchungen und Behandlungskosten reduziert. In einer regionalen Untersuchung im Südosten der USA gaben zudem 82 % der befragten Notfallmediziner an, dass sie wertvolle Zeit sparen konnten, wenn sie auf elektronische Informationen zur Patientenvorgeschichte zugreifen konnten – im Durchschnitt wurde die eingesparte Zeit auf 105 min geschätzt [13]. Patientenbefragungen aus den USA und Deutschland weisen darauf hin, dass auch die große Mehrheit der Patienten dem Austausch von Gesundheitsinformationen positiv gegenübersteht und bereit wäre, die eigenen Daten elektronisch verfügbar zu machen [14, 15].

Im Hinblick auf die vom Notaufnahmepersonal am dringendsten gewünschten Patienteninformationen zeigen sich auffallende Unterschiede zwischen der New Yorker Studie von Shapiro et al. [5] und den in diesem Artikel vorgestellten Befragungsergebnissen. So wurde Daten zu Medikamenteneinnahmen, Vorerkrankungen und Allergien hierzulande eine merklich höhere Priorität beigemessen, als dies in New York der Fall war, wo wiederum EKGs deutlich häufiger gewünscht wurden [5]. Die verschiedenen Ergebnisse der beiden Studien könnten neben den nationalen Unterschieden im Gesundheitssystem zum Teil auch durch den zeitlichen Abstand zwischen den Befragungen sowie durch eine andere Zusammensetzung der Studienpopulation bedingt sein. So wurden in die New Yorker Studie nicht das gesamte Notaufnahmepersonal, sondern lediglich die dort tätigen Ärzte einbezogen.

### Limitationen

Da kein Verteiler verfügbar ist, über den das gesamte deutsche Notaufnahmepersonal adressiert werden kann, wurde mit dem Verteiler der DGINA auf einen E‑Mail-Verteiler zurückgegriffen, der zwar verschiedene in der Notfallversorgung tätige Berufsgruppen und Fachdisziplinen abdeckt, jedoch keine repräsentative Auswahl darstellt. Wie aus den Angaben zur beruflichen Funktion der Befragten hervorgeht, sind sowohl unter den teilnehmenden Ärzten als auch unter den teilnehmenden Pflegekräften Personen in leitenden Positionen überproportional häufig vertreten. Zur Altersverteilung und zur regionalen Verteilung der Befragungsteilnehmer können keine Aussagen getroffen werden, da entsprechende Angaben zur Gewährleistung der Anonymität nicht erhoben wurden.

Als weitere Limitation zu benennen ist, dass eine exakte Bestimmung der Responserate nicht möglich ist, da der Verteiler der DGINA ebenso wie die Gruppe der Young DGINA auch eine nicht genau bekannte Anzahl von Personen umfasst, die ausschließlich in der präklinischen Notfallversorgung tätig ist und damit nicht zur Zielgruppe dieser Befragung zählt. In diesem Umstand ist zugleich auch ein wesentlicher Grund zu vermuten, weshalb viele Personen lediglich die Startseite des Fragebogens aufgerufen haben (von den 191 Personen, die den Fragebogen aufgerufen, aber nicht oder nicht vollständig ausgefüllt haben, sind nur 34 bis zur zweiten Seite oder weiter gelangt).

## Ausblick

Die Ergebnisse der hier durchgeführten Befragung spiegeln die aktuellen Herausforderungen, mit denen sich das Notaufnahmepersonal in Deutschland bei der Einholung von Informationen zur Patientenvorgeschichte konfrontiert sieht, wider. Wenn es gelingt, mit dem Ausbau der TI Fachanwendungen wie den Notfalldatensatz in Deutschland flächendeckend verfügbar zu machen, kann sich das Bild schon in absehbarer Zeit merklich verändern. Zudem könnten aktuelle Förderinstrumente wie beispielsweise das Krankenhauszukunftsgesetz für einen deutlichen Schub bei der Digitalisierung in deutschen Krankenhäusern sorgen. Die Auswirkungen dieser Entwicklungen auf die Verfügbarkeit von Patienteninformationen in der klinischen Notfallversorgung sollte Gegenstand weiterer Forschung sein. Hierbei sollte auch betrachtet werden, welchen Einfluss das Vorhandensein eines Notfalldatensatzes auf die Diagnose- und Therapiefindung hat – auch unter Beachtung möglicher systematischer kognitiver Fehler wie des sogenannten Diagnosis momentum, welches eine ungeprüfte Übernahme einer Vordiagnose durch den weiterbehandelnden Arzt kennzeichnet [16].

## Fazit für die Praxis

Im Zuge des Ausbaus der TI sollte insbesondere die Verfügbarkeit von NFD und DPE zügig ausgeweitet werden. Dadurch könnte der Zugang zu vielen von den Befragten als für die Notfallversorgung besonders relevant erachteten Daten wie Medikation, Vorbefunde, Allergien, Willenserklärungen und Kontaktdaten erheblich erleichtert werden. Ergänzend sollten im Rahmen der geplanten ePA auch Entlassungsbriefe sowie Röntgen- und andere Bilddaten elektronisch verfügbar gemacht werden, da diese aufgrund des limitierten Speicherplatzes der eGK nicht Teil des NFD sein können, von vielen Befragten aber ebenfalls als sehr wichtig eingestuft wurden. Wie die Befragungsergebnisse zur Wahrnehmung der medizinischen Fachanwendungen der TI nahelegen, sollte neben der technischen Verfügbarkeit außerdem auch die Bekanntheit dieser Anwendungen innerhalb des Notaufnahmepersonals u. a. durch Informationskampagnen erhöht werden.
